# The Relation Between Histological, Tumor-Biological and Clinical Parameters in Deep and Superficial Leiomyosarcoma and Leiomyoma

**DOI:** 10.1080/1357714021000065404

**Published:** 2002-09

**Authors:** Justin Pijpe, Gerben H. Torn Broers, Boudewijn E.Ch. Plaat, M. Hundeiker, F. Otto, Mirjam F. Mastik, Harald J. Hoekstra, Winette T. A. van der Graaf, Eva van Den Berg, Willemina M. Molenaar

**Affiliations:** 1 Department of Pathology University Hospital of Groningen Groningen The Netherlands; 2 Fachklinik Hornheide Münster Germany; 3 Department of Surgical Oncology University Hospital of Groningen Groningen The Netherlands; 4 Department of Medical Oncology University Hospital of Groningen Groningen The Netherlands; 5 Department of Medical Genetics University Hospital of Groningen Groningen The Netherlands; 6 Department of Oral and Maxillofacial Surgery University Hospital Groningen PO Box 30.001 Groningen 9700 RB The Netherlands

## Abstract

*Purpose:* Leiomyosarcomas (LMS) of deep and superficial tissues were examined to identify prognostic markers explaining their different biological behaviour and to define differences between cutaneous and subcutaneous LMS. LMS and leiomyomas (LM) of the skin were compared to and consistent differences that could aid in the (sometimes difficult) diagnosis.

*Patients:* Material was obtained from 27 patients with a deep LMS, 14 with a superficial LMS, and 21 with a LM.

*Methods:* Proliferation markers (mitotic and Ki-67 indices), DNA ploidy, size, grade, and the amount of apoptosis were studied. Statistical analysis was performed and survival curves were constructed by the Kaplan-Meier method and compared by the log-rank test.

*Results:* Superficial LMS were smaller than deep LMS (*p* < 0.05), and the overall survival of patients with a superficial LMS was better than with a deep LMS (*p* < 0.05).Within the group of superficial LMS only entirely subcutaneous, and not cutaneous tumors metastasized.No differences were found in the other examined parameters. Proliferation and apoptotic indices were significantly higher in superficial LMS compared to superficial LM.

*Discussion:* The difference in clinical outcome between patients with a superficial and deep LMS, seems to be related to site and size.The metastatic potential of subcutaneous LMS, however, seems to be related to location alone and not to size.The amount of apoptosis and proliferation can be used as additional criteria in the differentiation between superficial LMS and LM.


*Sarcoma* (2002) **6**, 105–110

Taylor & Francis healthsciences

**ORIGINAL ARTICLE**

### The relation between histological, tumor-biological and clinical parameters in deep and superficial leiomyosarcoma and leiomyoma

JUSTIN PIJPE[1], GERBEN H. TORN BROERS[1], BOUDEWIJN E.CH. PLAAT[1], M. HUNDEIKER[2], F. OTTO[2], MIRJAM F. MASTIK[1], HARALD J. HOEKSTRA[3], WINETTE T.A. VAN DER GRAAF[4], EVA VAN DEN BERG[5] & WILLEMINA M. MOLENAAR[1]

*Departments of [1]Pathology, [3]Surgical Oncology, [4]Medical Oncology and [5]Medical Genetics, University Hospital of Groningen, Groningen, The Netherlands, [2]Fachklinik Hornheide, Münster, Germany*

**Abstract**

*Purpose:* Leiomyosarcomas (LMS) of deep and superficial tissues were examined to identify prognostic markers explaining their different biological behaviour and to define differences between cutaneous and subcutaneous LMS. LMS and leiomyomas (LM) of the skin were compared to find consistent differences that could aid in the (sometimes difficult) diagnosis.

*Patients:* Material was obtained from 27 patients with a deep LMS, 14 with a superficial LMS, and 21 with a LM.

*Methods:* Proliferation markers (mitotic and Ki-67 indices), DNA ploidy, size, grade, and the amount of apoptosis were studied. Statistical analysis was performed and survival curves were constructed by the Kaplan-Meier method and compared by the log-rank test.

*Results:* Superficial LMS were smaller than deep LMS ($p < 0.05$), and the overall survival of patients with a superficial LMS was better than with a deep LMS ($p < 0.05$). Within the group of superficial LMS only entirely subcutaneous, and not cutaneous tumors metastasized. No differences were found in the other examined parameters. Proliferation and apoptotic indices were significantly higher in superficial LMS compared to superficial LM.

*Discussion:* The difference in clinical outcome between patients with a superficial and deep LMS, seems to be related to site and size. The metastatic potential of subcutaneous LMS, however, seems to be related to location alone and not to size. The amount of apoptosis and proliferation can be used as additional criteria in the differentiation between superficial LMS and LM.

### Introduction

Leiomyosarcomas (LMS) are relatively uncommon and account for 7% of soft tissue sarcomas (STS). Just as their benign counterparts, leiomyomas (LM), they show differentiation towards smooth muscle.[1] Few studies have focused exclusively on this rare type of tumor. In STS, tumor grade is considered to be the most significant prognostic factor, the two most important parameters being the number of mitotic figures and the extent of necrosis.[1] Various studies[2–5] examined the prognostic value of other parameters, such as DNA ploidy, Ki-67 (a monoclonal antibody, which reacts with a nuclear antigen expressed in all phases of the cell cycle except $G_0$), and apoptosis, in heterogeneous groups of STS patients. Overall, aneuploidy, a high amount of Ki-67, and low frequencies of apoptotic cells seem to be related to a worse clinical outcome in patients with different types of STS. The significance of the above-mentioned parameters may differ among various histological types of STS, which may be the reason for inconsistent results between different studies.[6,7] Studies focusing exclusively on LMS, express the significance of the site of the tumor on its behaviour, i.e., the prognosis of patients with (sub)cutaneous LMS is better than that of patients with deep-seated LMS, despite their similar histological and tumor biological features.[1,8–11] Jensen *et al.* recommended that superficial LMS be separated further into lesions with predominant cutaneous growth and tumors that involve the subcutis, since cutaneous LMS generally have a better prognosis, and only tumors involving the subcutis have the tendency to metastasize.[1,10–13]

Correspondence to: J. Pijpe, M.D., Department of Oral and Maxillofacial Surgery, University Hospital Groningen, PO Box 30.001, 9700 RB Groningen, The Netherlands. Fax: +31-50-361-1136; E-mail: j.pijpe@kchir.azg.nl

Partly presented in poster form at the Annual Meeting of the American Society of Clinical Oncology, 18 May 1999, Atlanta, GA, USA.

ISSN 1357–714X print/ISSN 1369–1643 online/02/0200105–06 © Taylor & Francis Ltd
DOI: 10.1080/1357714021000065404

In the diagnosis of smooth muscle tumors, the distinction between malignant and benign may be difficult.[1] Factors which discriminate most in the clinical differential diagnosis are tumor size and location, benign tumors usually being small and located superficially.[14] To date, no study has compared (sub)cutaneous LMS with (sub)cutaneous LM.

The aim of this study is to find possible differences between LMS of deep soft tissue (excluding gastrointestinal stromal tumors and urogenital tumors), subcutaneous and cutaneous tissue, and to determine whether proliferation markers (mitotic and Ki-67 indices), size, grade, DNA ploidy and the amount of apoptosis correlate with the differences in their biological and clinical behaviour. We also analysed differences between LMS and LM of the cutis and subcutis, in order to find new diagnostic criteria.

### Patients

We collected all available material of all patients diagnosed between 1980 and 1998 with a LMS or a superficial LM in the northern region of The Netherlands. Material of 16 patients with superficial LM(S) was donated by the Department of Dermatopathology, Fachklinik Hornheide in Germany. The diagnosis was based on the criteria of Enzinger and Weiss,[1] using light microscopic examination of hematoxylin-eosin (HE)-stained paraffin sections, and, when necessary, the diagnosis was confirmed immunohistochemically using antibodies to actin and desmin. A total of 62 patients were included in the present study.

Material was obtained from 27 patients with a primary LMS of deep soft tissue (Table 1). The median age was 60 (range 20–83) years. Thirteen patients had extremity tumors, whereas three patients had a retroperitoneal LMS.

Fourteen patients with a superficial LMS (Table 1) were included in this study. The median age was 62 (range 23–85) years. Three patients had a tumor confined to the cutis, whereas seven patients had a superficial LMS involving both the cutis and the subcutis. The remaining four patients had a tumor confined to the subcutis.

Twenty-one patients with a superficial LM (Table 1) were analysed. Except for one, all tumors were confined to the cutis, so no distinction was made between cutaneous or subcutaneous tumors. The median age was 56 (range 7–83) years. A tumor was considered a LM, when no mitoses were found.

The patient records were used to collect the clinical data. Overall survival (OS) and survival status (NED, no evidence of disease; AWD, alive with disease; DOD, died of disease; DOC, died of other causes; LOF, lost to follow-up) were recorded for the patients with a deep and superficial LMS. Recurrence and metastatic disease were recorded as well (Table 1).

### Methods

### *Grading*

The LMS were graded according to the system described by Coindre *et al.*,[15] in which points are assigned to differentiation level (1, closely resembling normal tissue; 2, certain histogenetic classification; 3, undifferentiated), mitotic index per 2 $mm^2$ (1, 0–9; 2, 10–19; 3, 20 or more) and necrosis (0, none; 1, less than 50%; 2, more than 50%). Tumors with a total score of 2 or 3 were graded as grade I, those with a total score of 4 or 5 as grade II and those with a total score of 6–8 as grade III.

### *DNA ploidy*

DNA flow cytometry was performed on single cell suspensions obtained from formalin-fixed paraffin-embedded tissue or fresh tissue, as previously described by Plaat *et al.*[6] The DNA profile was considered (near)diploid when a single stem line was present in the diploid range; all others were considered aneuploid.

### *Proliferation and apoptosis*

For proliferation, the monoclonal antibody MIB 1, which recognizes the Ki-67 antigen, was used (Immunotech S.A., Marseille, France). Immunohistochemistry was performed on paraffin sections (4 μm) according to a method modified from Shi *et al.*[16,17]

Apoptosis was studied in 4-μm sections of formaldehyde-fixed and paraffin-embedded tissue using the TUNEL (terminal deoxynucleotidyl transferase (TdT) mediated dUTP nick end labeling) method, as previously described by Plaat *et al.*[18]

### *Quantification of Ki-67 and apoptosis*

For measuring the Ki-67 labeling index and the apoptotic labeling index we used ocular micrometry on a Leitz microscope by using an eyepiece grid at ×400 magnification. Fifteen fields were randomly selected throughout histologically viable areas. The positive and negative nuclei were counted. Endothelial cells, inflammatory cells and necrosis were excluded. The number of positive nuclei was then divided by the total number of nuclei in each of the fifteen randomly selected fields to calculate an index per field. The Ki-67 and the apoptotic indices were defined as the mean of the indices of the 15 randomly selected fields.

### *Statistical analysis*

To compare OS and survival status in relation to grade, size, DNA ploidy, and Ki-67 and apoptotic indices, survival curves were constructed by the

**Table 1.** *Patients with deep seated LMS, superficial LMS and LM (62 patients)*

| Location | *N* | Age (years) | Sex | Metastasis/ Local recurrence | Follow-up (whole group) (status and duration) |
|---|---|---|---|---|---|
| *Deep LMS* | 27 | 20–83 (median: 60) | 11 M/16F | | 9 NED (median: 33 months)<br>3 AWD (median: 23 months)<br>14 DOD (median: 39 months)<br>1 DOC (151 months) |
| Retroperitoneal | 3 | | | 2 Metastases | |
| Extremities | 13 | | | 5 Metastases, 2 local recurrences, 1? | |
| Other | 9 | | | 3 Metastases, 1 local recurrence, 2? | |
| *Superficial LMS* | 14 | 23–85 (median: 62) | 9 M/5F | | 11 NED (median: 26 months)<br>2 AWD (median: 18 months)<br>1 LOF |
| Cutaneous | 3 | | | No | |
| Cutaneous/ subcutaneous | 7 | | | 2 local recurrences | |
| Subcutaneous | 4 | | | 3 Metastases | |
| *Superficial LM* | 21 | 7–83 (median: 56) | 8 M/13F | None | N.A. |

(M, male; F, female; N.A., not available; NED, no evidence of disease; AWD, alive with disease; DOD, died of disease; DOC, died of other cause; LOF, lost to follow-up; ?, unknown)

Kaplan-Meier method. Survival curves in the different groups were compared by the log-rank test. A $\mu^2$-test or $\mu^2$-test for trend was used to estimate the differences in tumor grade and DNA ploidy. A Mann-Whitney *U*-test was used to analyse the differences between proliferation markers (mitoses, Ki-67), apoptosis and size in the different groups. A *p* value of $<0.05$ was considered statistically significant.

### Results

The results of the different groups are shown in Table 2. Due to technical failures and in some cases to insufficient material, not all tumors could be analysed for Ki-67 and apoptotic indices, grading, or DNA ploidy. By analyzing the total group of 41 patients with a LMS, no significant correlations were found between OS and mitotic, Ki-67 indices, apoptotic indices, grade, or DNA ploidy (Table 2).

### *Deep versus superficial LMS*

Patients with a superficial LMS had a significantly better OS compared to patients with a deep LMS (Fig. 1). More than half of all patients with a deep LMS died of the tumor (52%). This is in contrast with superficial LMS, where none of the patients died of the tumor. No differences were found between deep and superficial LMS for mitotic, Ki-67 or apoptotic indices, nor for grade and DNA ploidy (Table 2). Superficial LMS had a significantly ($p = 0.001$) smaller size than deep LMS; 2.5 vs. 9.5 cm (Fig. 2).

### *Cutaneous versus subcutaneous LMS*

Of the 14 superficial LMS, three tumors were limited to the cutis, four to the subcutis, and seven tumors involved both the cutis and the subcutis (Table 1). None of the three entirely cutaneous LMS metastasized, whereas three of the four subcutaneous tumors metastasized. None of the seven cutaneous/subcutaneous tumors metastasized, although two of them showed a local recurrence ($p > 0.5$). No statistical differences were found between mitotic, Ki-67 and apoptotic indices, grade, and DNA ploidy between the three groups (Table 2).

### *Superficial LM versus superficial LMS*

The superficial LMS had a mean Ki-67 index of 8.4% and a mean apoptotic index of 0.33%. In the LM this was 1.0 and 0.01%, respectively ($p < 0.005$) (Figs. 3 and 4). Two superficial LMS were diploid, two were aneuploid. All of the LM were diploid ($p = 0.19$).

### Discussion

The significance of various prognostic factors, such as DNA ploidy, Ki-67 and apoptotic indices, may differ among various types of STS.[2–7] Most studies include only a few smooth muscle tumors, or compare different types, so that interpretation of the results is not always easy. LMS is a rare tumor, and its behaviour seems to depend on the site of the tumor.[8–11] Therefore we studied LMS of deep and superficial tissue as one group to identify site-independent prognostic markers, which could explain the differences in biological behaviour. LMS and LM of the skin were also compared to find consistent differences that could aid in the differential diagnosis of malignant and benign superficial smooth muscle tumors.

Forty-one patients with a deep or superficial LMS were analysed in order to find factors that could predict clinical outcome. Although studies examining

**Table 2.** *Results of all LMS, deep LMS, superficial LMS and LM*

| | All LMS (41)* | Deep LMS (27)* | Superficial MS (14)* | leiomyoma (21)* | Deep vs superficial LMS | Superficial LMS vs LM |
|---|---|---|---|---|---|---|
| *Grade (n):* | | | | | $p > 0.05$ | – |
| I | 13 (34%) | 8 (31%) | 5 (42%) | – | | |
| II | 17 (45%) | 11 (42%) | 6 (50%) | – | | |
| III | 8 (21%) | 7 (27%) | 1 (8%) | – | | |
| *Size (cm)* | | | | | $p = 0.001$ | – |
| *n* | 34 | 23 | 11 | – | | |
| Range | 0.05–30 | 2.0–30.0 | 0.5–11 | | | |
| Mean; median | 8; 7.0 | 10; 9.5 | 3.6; 2.5 | | | |
| *Mitotic index* (number of mitoses per 2 mm$^2$ | | | | | $p > 0.05$ | – |
| *n* | 38 | 26 | 12 | | | |
| Range | 0–44 | 1–44 | 0–25 | | | |
| Mean; median | 12; 9 | 13; 9 | 9; 8 | | | |
| Ki-67 index *(%)* | | | | | $p > 0.05$ | $p < 0.005$ |
| *n* | 37 | 24 | 13 | 18 | | |
| Range | 0.60–36.8 | 0.8–36.8 | 0.6–20.2 | 0.4–1.9 | | |
| Mean; median | 10.4; 9.2 | 11.5; 9.7 | 8.4; 8.1 | 1.0; 0.9 | | |
| *Apoptotic index (%)* | | | | | $p > 0.05$ | $p < 0.005$ |
| *n* | 36 | 23 | 13 | 17 | | |
| Range | 0.00–1.27 | 0.02–1.27 | 0.00–0.84 | 0.00–012 | | |
| Mean; median | 0.41; 0.32 | 0.46; 0.41 | 0.33; 0.27 | 0.01; 0.00 | | |
| *Diploid/ aneuploid* (% of analysed cases) | 8 (42%)/ 11 (58%) | 7 (44%)/ 9 (56%) | 1 (33%)/ 2 (67%) | 5 (100%)/0 | $p > 0.05$ | $p > 0.05$ |
| *OS-time (months)* | | | | | $p > 0.05$ | – |
| *n* = | 40 | 27 | 13 | – | | |
| Range | 4–151 | 4–151 | 5–101 | | | |
| Mean; median | 43; 27 | 47; 38 | 35; 23 | | | |
| *Died of tumor* | 14 (35%) | 14 (52%) | 0 | 0 | $p < 0.005$ | |

*Due to technical failures. not all tumors could be analysed for Ki-67 amd apoptotic indices, grading or DNA ploidy.

large groups of heterogeneous STS showed a relation between a high Ki-67 index and malignancy,[2] low apoptotic index and low grade,[3] and aneuploidy and bad prognosis,[4,5] we did not confirm any of these findings. This is similar to the findings of Gustafson *et al.*,[19] who did not find any relationship between DNA ploidy and prognosis either. Although in our study patients with a high-grade LMS did have a worse prognosis, this was not statistically significant, which may be due to the limited number of LMS studied.

The prognosis of the patients with a superficial LMS was significantly better than that of patients with a deep LMS, which is in agreement with other studies.[1,8] We found no difference in proliferation markers (Ki-67 and mitotic indices), DNA ploidy and the amount of apoptosis. In a previous study, deep seated LMS with a high amount of Ki-67 tended to have a worse prognosis, but this was also not significant.[8] Although there seems to be a difference in grade between the two groups, i.e. deep LMS showing more high-grade tumors than superficial LMS (27 against 8%), this was not statistically significant. The different biological behaviour of these two types of tumors, seems to be only associated with their different locations. This is similar to a previous study, where LMS in superficial and deep soft tissues were almost the same with regard to cell proliferation and alteration of the *p53* gene.[8] However, we found a significantly different size between the two groups (deep LMS being larger than superficial LMS), which also could explain the different outcome. It seems plausible that superficial LMS are earlier discovered, so that their growth is limited. The conclusion is that the different outcome seems to be related only to the location and size, the latter probably being the most important.

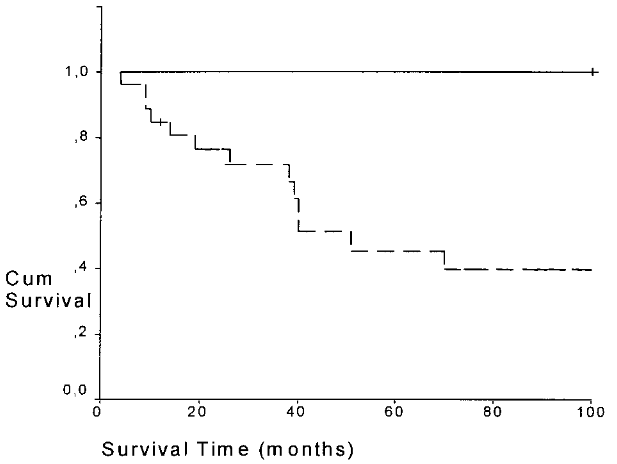

**Fig. 1.** *Overall survival curve: deep and superficial leiomyosarcoma (LMS). Solid line, superficial LMS; dotted line, deep LMS. p < 0.05.*

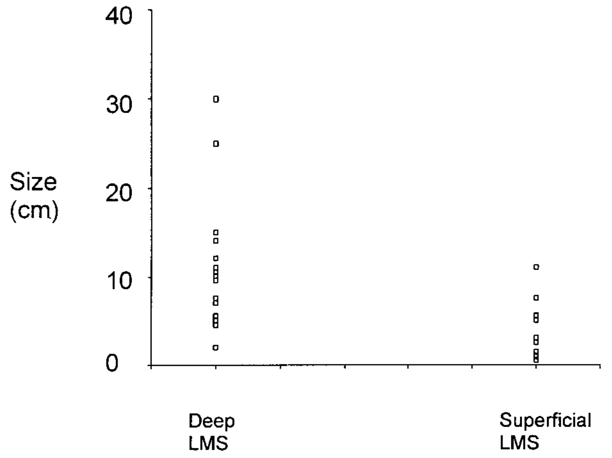

**Fig. 2.** *Deep and superficial leiomyosarcoma (LMS): size. p = 0.001.*

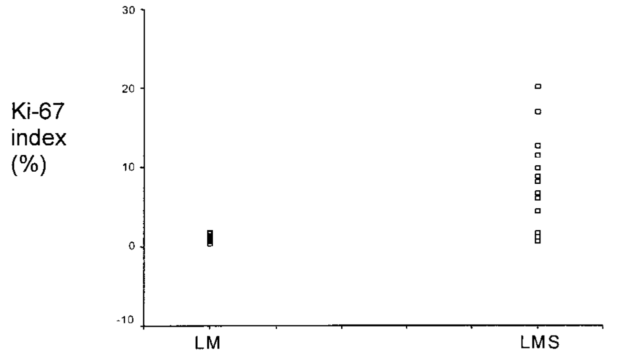

**Fig. 3.** *Leiomyoma (LM) and superficial leiomyosarcoma (LMS): Ki-67 indices. p < 0.005.*

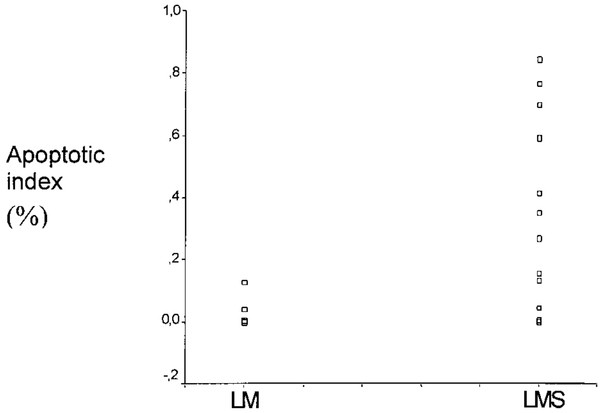

**Fig. 4.** *Leiomyoma (LM) and superficial leiomyosarcoma (LMS): apoptotic indices. p < 0.005.*

Within the group of superficial LMS, site seems also to be of importance in cutaneous and subcutaneous tumors, as they have a different prognosis. Tumors confined to the cutis seem to have a better clinical outcome.[1,10,11] Identifying patients with a greater risk of metastasis is important to determine the prognosis or therapy. In agreement with other studies,[11–13] only tumors confined to the subcutis metastasized. We found that patients with a LMS confined to the cutis, did not show metastasis or recurrences. Proliferation and apoptosis did not predict metastatic potential in superficial LMS, nor did any of the other parameters. Although in one study only patients with diploid superficial LMS had metastases,[20] we did not find any relation between malignancy and DNA ploidy. Analyses of larger groups maybe necessary to examine the relevance of DNA ploidy. The impaired prognosis might be related to location alone, contrary to deep and superficial LMS where size may also be of importance. Patients with a tumor involving both the cutis and the subcutis did not metastasize, but some of the patients had a local recurrence. It seems that this last group is of dermal origin, with invasion of the subcutis. Only tumors arising in the subcutis tend to metastasize; whether these two groups are different must be further examined. One explanation might be the difference in origin; the cutaneous LMS are usually arising from pilar arecti, and subcutaneous LMS are mostly from vascular origin.[21]

As mentioned above, differentiation between LM and LMS may be difficult. In rare cases, diagnosis can be hard, imposing difficulties upon the choice of the right therapy and expected clinical behaviour.[1] In agreement with one previous study,[22] comparing different types of LM and LMS, the amount of apoptosis and proliferation were significantly higher in the superficial LMS compared to the LM. This is in contrast with another study,[3] describing heterogeneous groups of STS, where less apoptosis is associated with a worse outcome. This suggests a type-specific phenomenon. Apoptosis and proliferation can be used as additional criteria in the differentiation between LMS and LM of the skin. DNA ploidy may also be of diagnostic importance, all of the LM being diploid, but this should be confirmed in a larger series of tumor samples. It may be possible that these results also apply to other types of malignant and benign smooth muscle tumors, and might aid in the diagnosis of difficult cases.

In summary, our study shows that the clinical behaviour of different types of LMS seems to be related to the site of the tumor alone. The smaller tumor size in patients with a superficial LMS, compared to deep, is probably due to the early discovery of this kind of LMS, and may also be the main reason for the better clinical outcome of these patients. The reason why subcutaneous LMS have a worse prognosis than entirely cutaneous LMS is not yet clear, but might be related to the tumor origin. Further examination is necessary to find out of these types of LMS have a different oncogenesis, which may account for their different behaviour. Difficulties in the diagnosis between superficial LMS and LM, can be facilitated by determination of the amount of apoptosis and proliferation.

### References

1. Weiss SW, Goldblum JR. *Enzinger and Weiss's soft tissue tumors*. 4th Ed. St Louis, MO: C.V. Mosby, 2001.
2. Ueda T, Aozasa K, Tsjujimoto M, *et al.* Prognostic significance of Ki-67 reactivity in soft tissue sarcomas. *Cancer* 1989; 63: 1607–11.
3. Nakanashi H, Ohsawa M, Naka N, Uchida A, Ochi T, Aozasa K. Immunohistochemical detection of bcl-2 and p53 proteins and apoptosis in soft tissue sarcoma: their correlations with prognosis. *Oncology* 1997; 54: 238–44.
4. Agarwal V, Greenebaum E, Wersto R, Koss LG. DNA ploidy of spindle cell soft-tissue tumors and its relationship to histology and clinical outcome. *Arch Pathol Lab Med* 1991; 115: 558–62.
5. Algevard TA, Berg NO, Baldetorp B, *et al.* Cellular DNA content and prognosis of high-grade soft tissue sarcoma: the Scandinavian Sarcoma Group experience. *J Clin Oncol* 1990; 8: 538–47.
6. Plaat BEC, Muntinghe FLH, Molenaar WM, *et al.* Clinical outcome of patients with previously untreated soft tissue sarcomas in relation to tumor grade, DNA ploidy and karyotype. *Int J Cancer* 1997; 74: 396–402.
7. Kuratsu S, Tomita Y, Myoui A, Uchida A, Ono K, Aozasa K. DNA ploidy pattern and cell cycle stage of tumor cells in soft-tissue sarcomas: clinical implications. *Oncology* 1995; 52: 363–70.
8. Konomoto T, Fukada T, Hayashi K, Kumazawa J, Tsuneyoshi M. Leiomyosarcoma in soft tissue: examination of p53 status and cell proliferating factors in different locations. *Hum Pathol* 1998; 29(1): 74–81.
9. Hashimoto H, Daimaru Y, Tsuneyoshi M, *et al.* Leiomyosarcoma of the external soft tissue. *Cancer* 1986; 57: 2077–88.
10. Jensen ML, Jensen OM, Michalski W, Nielsen OS, Keller J. Intradermal and subcutaneous leiomyosarcoma: a clinicopathological and immunohistochemical study of 41 cases. *J Cutan Pathol* 1996; 23: 458–63.
11. Fields JP, Helwig EB. Leiomyosarcoma of the skin and subcutaneous tissue. *Cancer* 1981; 47(1): 156–69.
12. Spencer JM, Amonette RA. Tumors with smooth muscle differentiation. *Dermatol Surg* 1996; 22: 761–8.
13. Dahl I, Angervall L. Cutaneous and subcutaneous leiomyosarcoma: a clinicopathological study of 47 patients. *Pathol Eur* 1974; 9(4): 307–15.
14. Myhre-Jensen O. A consecutive 7-year series of 1331 benign soft tissue tumours. *Acta Orthop Scand* 1981; 52: 287–93.
15. Coindre JM, Trojani M, Contesso G, *et al.* Reproducibility of a histopathologic grading system for adult soft tissue sarcoma. *Cancer* 1986; 58: 306–9.
16. Shi SR, Key ME, Kalra KL. Antigen retrieval in formalin-fixed, paraffin-embedded tisues: an enhancement method for immunohistochemical staining based on microwave oven heating of tissue sections. *J Histochem Cytochem* 1991; 39: 741–8.
17. Emanuels A, Hollema H, Koudstaal J. Autoclave heating: an alternative method for microwaving? *Eur J Morph* 1994; 32: 337–40.
18. Plaat BEC, Molenaar WM, Mastik MF, Koudstaal J, Van den Berg E, Schraffordt Koops H, Hoekstra HJ. Hyperthermic isolated limb perfusion with TNF-( and melphalan in patients with locally advanced soft tissue sarcomas: treatment response and clinical outcome related to changes in proliferation and apoptosis. *Clin Cancer Res* 1999; 5(7): 1650–7.
19. Gustafson P, Willén, Baltedorp B, *et al.* Soft tissue leiomyosarcoma. A population-based epidemiologic and prognostic study of 48 patients, including cellular DNA content. *Cancer* 1992; 70(1): 114–8.
20. Oliver GF, Reiman HM, Gonchoroff NJ, Muller SA, Umbert IJ. Cutaneous and subcutaneous leiomyosarcoma: a clinicopathological review of 14 cases with reference to antidesmin staining and nuclear DNA patterns studied by flow cytometry. *Br J Dermatol* 1991; 124(3): 252–7.
21. Farshid G, Pradhan M, Goldblum J, Weiss SW. Leiomyosarcoma of somatic soft tissues: a tumor of vascular origin with multivariate analysis of outcome in 42 cases. *Am J Surg Pathol* 2002; 26(1): 14–24.
22. Valenti MT, Azzarello G, Vinante O, *et al.* Differentiation, proliferation and apoptosis levels in human leiomyoma and leiomyosarcoma. *J Cancer Res Clin Oncol* 1998; 124: 93–105.
